# Ectopically Expressed Perforin-1 Is Proapoptotic in Tumor Cell Lines by Increasing Caspase-3 Activity and the Nuclear Translocation of Cytochrome c

**DOI:** 10.1371/journal.pone.0040639

**Published:** 2012-07-19

**Authors:** Li-Feng Wang, Fang Wang, Jun-Tang Li, Wei-Hong Wen, Jing Zhao, Lin-Tao Jia, Yan-Ling Meng, Yun-Xin Cao, Li-Bo Yao, Si-Yi Chen, Yan-Ming Xu, An-Gang Yang

**Affiliations:** 1 State Key Laboratory of Cancer Biology, Departments of Biochemistry and Molecular Biology, School of Basic Medical Sciences, Fourth Military Medical University, Xi’an, China; 2 Department of Immunology, School of Basic Medical Sciences, Fourth Military Medical University, Xi’an, China; 3 Department of Microbiology, School of Basic Medical Sciences, Fourth Military Medical University, Xi’an, China; 4 Department of Molecular Microbiology and Immunology, Norris Comprehensive Cancer Center, Keck School of Medicine, University of Southern California, Los Angeles, California, United States of America; 5 Department of Cell Biology and Genetics, Shantou University Medical College, Shantou, China; University of Pecs Medical School, Hungary

## Abstract

Perforin-1 (PRF), a cytotoxic lymphocyte pore-forming protein, plays an important role in the action of cytotoxic T cells and natural killer cells in that it causes the lysis of abnormal body cells and the elimination of virus-infected cells and tumors. Upon degranulation, PRF inserts itself into the target cell’s plasma membrane, forming a pore. The subsequent translocation of pro-apoptotic granzymes (including granzyme B, A, M et al.) into the cytoplasm provides the proteases with access to numerous protein substrates that promote apoptosis after cleavage. These proteases are believed to be the main executioners of target cell apoptosis. Although the PRF and granzyme components are both critical to this process and in some way involved in inducing cell death in target cells, the inhibition of tumor growth could still be efficient in granzyme-deficient mice. It is unclear whether PRF alone can suppress tumors. In this study, we discovered that forced ectopic expression of PRF alone, in the absence of granzymes, could mediate cell death in cancer cells. Notably, transient expression of both full-length and truncated active-form PRF in human Hep G2, SK-BR-3, and HeLa cells was found to induce apparent cell growth inhibition and cell death, as evidenced by chromosome condensation and DNA fragmentation, increased caspase-3 activity, and the release of apoptosis inducing factor (AIF) and cytochrome *c* from the mitochondria. This PRF-induced cell death could be abrogated by pan-caspase inhibitor (Z-VAD) and mitochondria protector (TAT-BH4). The implication of these results is that ectopically expressed PRF has apoptosis-inducing abilities, and PRF alone is sufficient to induce apoptotic cell death in cells with ectopic expression. Taking this into consideration, our results suggest the possibility of using PRF as a pro-apoptotic gene for tumor therapeutics.

## Introduction

Perforin-1 (PRF), encoded by the *PRF* gene in humans, is a cytolytic protein found in the granules of CD8 T-cells, natural killer (NK) cells, and stimulated peripheral blood lymphocytes [1], [2]. Perforin plays an important role in CD8 T-cell and NK cell function for lysis of abnormal body cells which were bound by antibodies and subsequently recognized by NK cells. As an essential component of cytotoxic granules, perforin contributes to T cell-mediated death via apoptosis or necrosis as indicated by the permeabilization of target cell membranes [3], [4], [5]. Human perforin-1 is also called pore-forming protein, cytolysin, or complement C9-related protein because of its biological functions in cell lysis. It shares homology with terminal complement components. Moreover, like complement, PRF multimerizes in membranes to form pores [2], [6], [7], [8]. Human perforin-1 contains 534 amino acids preceded by a 21-residue leader peptide, which is typical of secreted and granule-stored protein, and three potential glycosylation sites. Perforin-1 is responsible for the Ca^2+^-dependent lytic activity of granules [9]. Upon degranulation, the active form of perforin exposes its calcium-dependent lipid binding C2 domain, and inserts itself into the target cell's plasma membrane, forming pores [10]. Its membrane attack complex/perforin domain exhibits lytic membrane-inserting ability [11]. It shares homology with cholesterol-dependent cytolysins from Gram-positive bacteria [12]. The subsequent translocation of pro-apoptotic granzymes into the cytoplasm provides the proteases with access to numerous protein substrates that promote apoptosis after cleavage [13], [14], [15], [16].

Perforin is the only known delivery molecule in the granule exocytosis pathway involved in mediating target-cell apoptosis, which is the main way by which cytotoxic T lymphocytes (CTL) and NK cells, collectively known as cytotoxic lymphocytes (CLs), eliminate virus-infected cells and tumors. Mice deficient in PRF are profoundly immunodeficient and are unable to protect themselves against viral infection and tumors [3]. And humans with familial hemophagocytic lymphohistiocytosis (HLH) due to PRF gene mutations also have compromised antiviral immunity [17].

Although PRF was identified more than 25 years ago, some of the molecular and cellular bases for PRF activity remain undefined [18], [19]. Recently, electron microscopy analysis has revealed that the PRF pores on the cell membrane are big enough for granzymes to pass through directly [10]. However, dynamic killing morphology has shown that PRF pores induced membrane repair response ultimately facilitated endocytosis-dependent granzyme uptake [20], [21]. On the other hand, the inhibition of tumor growth in granzyme-deficient mice has indicated that perforin might also work independently [22]. Pores formed during endosomal uptake process would facilitate the release of PRF monomers themselves into the cytosol, though there is a question as to whether these monomers still function in the cytoplasm. It is arousing to bring to light that the C terminus regulate trafficking of PRF during CLs exocytose [23]. It remains unknown whether any cellular mechanisms restrain this toxic protein as it traverses effectors’ organelles [24]. It is possible that this kind of restraint may depend solely on the presence of structurally intact PRFs. One recent study demonstrated that UV-B irradiation induced *PRF* gene and protein expression in keratinocytes, and the latter acquired cytotoxic potential against a variety of cellular skin targets, without causing any toxic response in the host cell themselves [25]. This may indicate that structurally intact PRF is sufficient for the self-protection of CLs and PRF-expressing hematopoietic cells.

In the present study, we examined several cancer cell lines by ectopically expressing PRF and demonstrated its intracellular toxicity in the host cell. Growth inhibition, cell cycle transition, phosphatidylserine residue exposure, chromosome condensation, and DNA fragmentation were observed. Increased caspase-3 activity and release of AIF and cytochrome *c* from the mitochondria further demonstrated that PRF alone might be sufficient to induce apoptotic cell death.

## Results

### Expression of Perforin Leads to Cancer Cell Death

**Figure 1:**
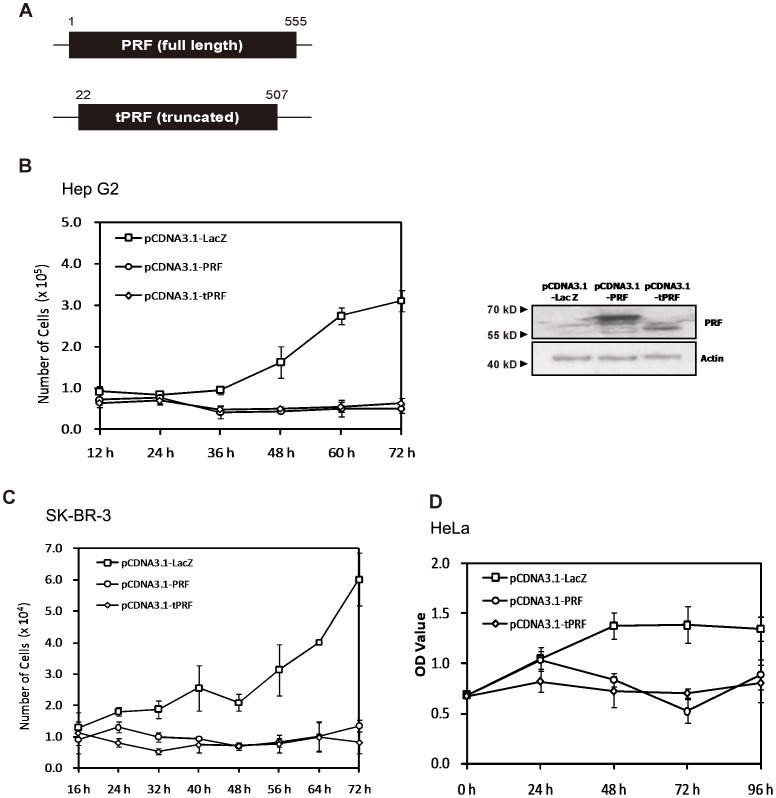
Role of perforin expression in cancer cell death. A) Full length and truncated perforin. Both the full-length and truncated perforin genes, PRF and tPRF, were obtained from IL-2 stimulated Jurkat cells through polymerase chain reaction and cloned in pCDNA3.1 vector. B–D) Growth curves of transfected Hep G2 (B, left) and SK-BR-3 cells (C) were assessed by cell counting with the mean data from three or more independent experiments, each conducted in triplicate. HeLa cells (D) were assessed by a MTT assay. Expressions of full-length and truncated perforin were determined using Western blot analysis (B, right).

To disclose the potency of cytosol translocalized perforin, we constructed a truncated perforin (Fig. 1A) in which the signal peptide was removed and the C2 domain was reserved as the C-terminal end, according to the NCBI database (Accession #: NP_005032.1; GI: 4826942) to generate an interior active form. Full length perforin was also constructed (Fig. 1A) to determine whether the intact structure would be toxic to nonhematopoietic host cancer cells.

As shown in Fig. 1B (left), in contrast to the LacZ-transfectants, transient expression of perforin genes in human hepatocellular carcinoma Hep G2 cell, both in full-length and truncated forms, led to apparent inhibition of cell growth from 36 h after transfection. Enhanced inhibition of cell growth and cell death were observed over a prolonged period of time. The expression levels of full-length and truncated perforin were confirmed by Western blot (right) in a paralleled transfected group (pCDNA3.1-LacZ, -PRF and -tPRF) 24 h after transfection. Similar growth inhibitions were obtained in human breast cancer SK-BR-3 (Fig. 1C) cell and human uterocervical carcinoma HeLa cell (Fig. 1D), but not in human lymphoblast Jurkat cells (Supporting Information: Fig. S1), from which the perforin cDNA was amplified.

### Ectopic Expression of Perforin Impaired Cell Cycle

**Figure 2:**
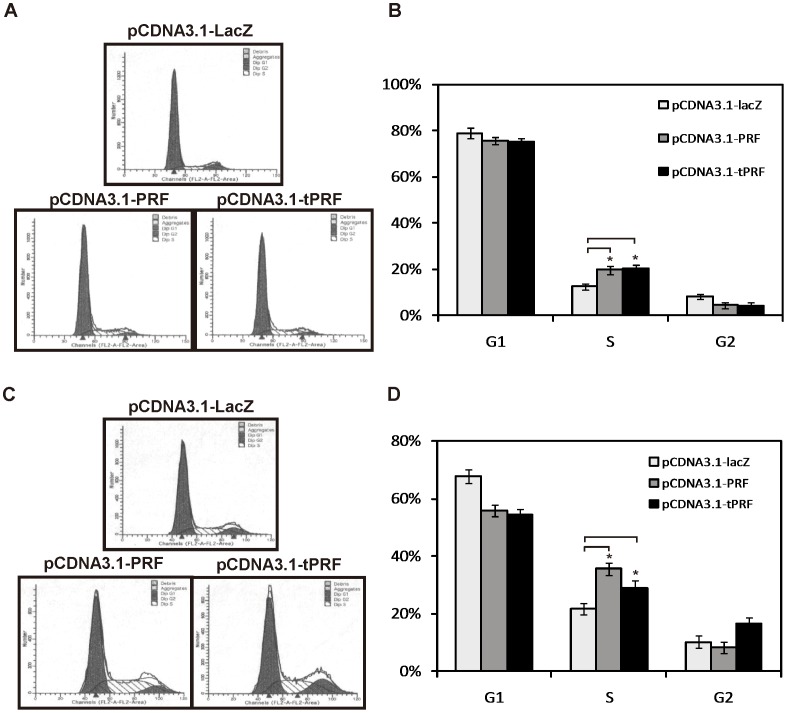
Effects of ectopic expression of perforin on the cell cycle. Twenty-four hours after transfection, SK-BR-3, and Hep G2 cells were digested and subjected to cell cycle determination. (A) Representative image of phase distribution of SK-BR-3 cell is presented. (B) The bar graph show the number of G1, S, and G2 phases and data are shown as mean ± S.D. of 3 experiments. (C) Phase distribution of Hep G2 cell was detected by flow cytometry and a representative image is presented. (D) Relative prevalence of G1, G2, and S phases among Hep G2 cell. Data are shown as mean ± S.D. of three experiments. *P* values were determined by unpaired Student’s t test (n = 3; * *P*<0.05).

Generally, changes in the cell cycle profile occurred when the cells showed an inhibited growth rate. To address the effect of perforin on the cell cycle transition, SK-BR-3 and HepG2 cells were both transfected with pCDNA3.1-PRF and pCDNA3.1-tPRF using pCDNA3.1-LacZ as controls, and cell cycle detections were performed by flow cytometric analysis. As shown in Figures 2B and 2D, which also representatively presented in Figures 2A and 2C, there were more perforin-expressing cells arrested in S phase than controls. No significant difference was observed between full-length and truncated groups.

### Cancer Cell Apoptosis Triggered by Perforin Transfection

**Figure 3:**
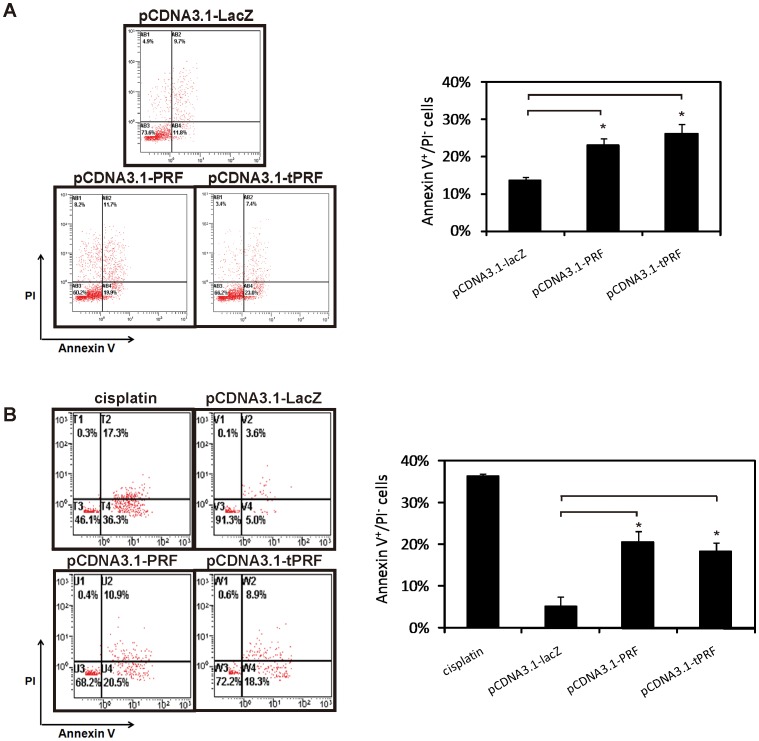
Cancer cell apoptosis triggered by perforin transfection. Twenty-four hours after transfection, (A) Hep G2 and (B) SK-BR-3 cells were digested and diluted into single cell suspension, stained by Annexin V/PI at 37°C for 10 min and measured by flow cytometry. Representative image of flow cytometry is shown (left panels). The bar graph (right) shows the number of Annexin V^+^/PI^-^ cells and data are shown as mean ± S.D. of 3 experiments. Data were analyzed with unpaired Student’s t test (n = 3; *P<0.05).

To determine what happens under the pressure of ectopic perforin expression, Annexin V binding assay coupled with PI staining was performed to detect the loss of cell membrane integrity and phospholipid asymmetry in Hep G2 (Fig. 3A) and SK-BR-3 (Fig. 3B) cells. In Figure 3, unlike the LacZ transfection groups, Annexin V positive, PI negative cells appeared to express more perforin (full-length and truncated), which demonstrated that cells expressing perforin were undergoing apoptosis. The apoptotic features detected by Annexin V indicated far more cytolytic activity from perforin than we had expected. More evidence is needed to determine the mechanisms underlying expressed perforin-induced cell death.

### Effects of Perforin Expression on Chromosome Condensation and DNA Fragmentation in Hep G2 and SK-BR-3 Cells

**Figure 4:**
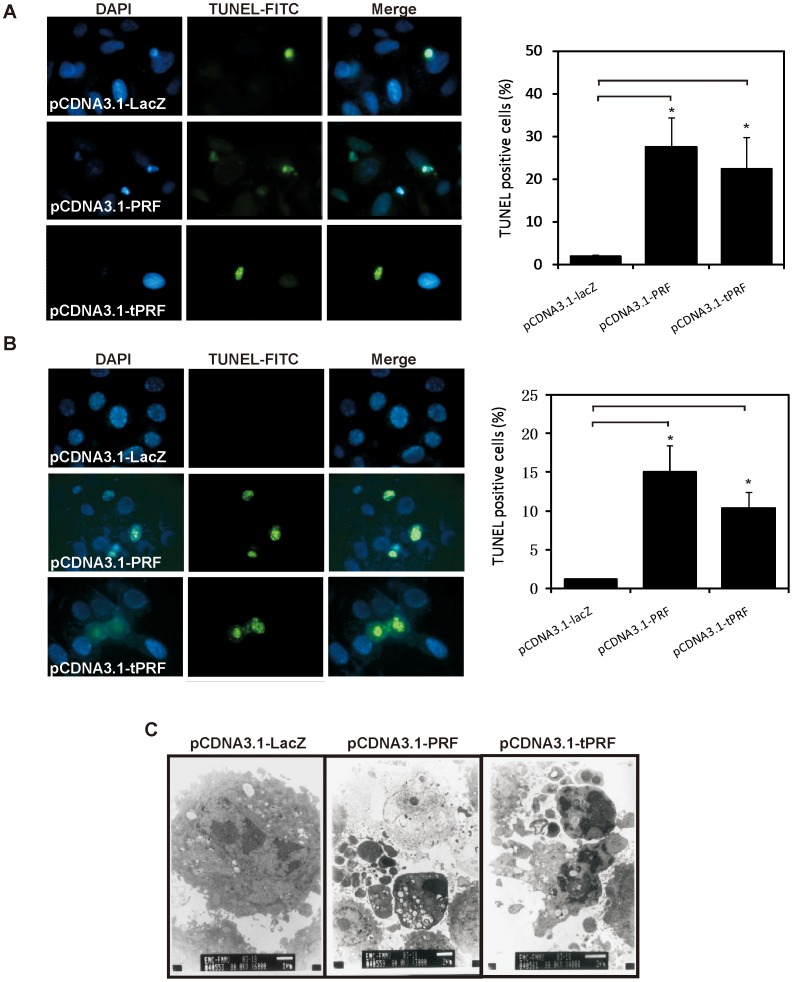
Effects of perforin expression on chromatin condensation and DNA fragmentation in Hep G2 and SK-BR-3 cell. (A) Hep G2 and (B) SK-BR-3 cells were labelled with Fluorescein FragEL™ DNA Fragmentation Detection Kit 24 hours post transfection and observed under a microscope. Representative photomicrograph of TUNEL-positive cells comparing LacZ control cells with perforin (both full length and truncated) overexpressing cells is shown (left panels), TUNEL positive cell is labeled by FITC (green), and the nuclei is labeled by DAPI (blue). The number of TUNEL-positive cells was determined by counting four independent fields (400× magnification) and the means of four determinations± SD are shown (right panels). Data were analyzed with unpaired Student’s t test (n = 4; * *P*<0.05). (C) Transmission electron microscopy of Hep G2 cells after transfection is also shown.

To confirm our observations regarding apoptotic cell death, TUNEL (TdT-Mediated X-dUTP Nick End Labeling) staining was performed to detect DNA cleavage. Significant increases in FITC labeled Hep G2 (Fig. 4A) and SK-BR-3 (Fig. 4B) cells indicated that perforin-transfected cells were undergoing apoptosis. In addition, as observed by electronic microscopy (Fig. 4C), the expression provoked typical apoptotic morphological changes, including chromatin condensation and its margination at the nuclear periphery, cellular shrinkage, and blebbing with intact plasma membranes.

### Effects of Perforin on Caspase-3 Activity and Release of AIF and Cytochrome *c* from Mitochondria

**Figure 5:**
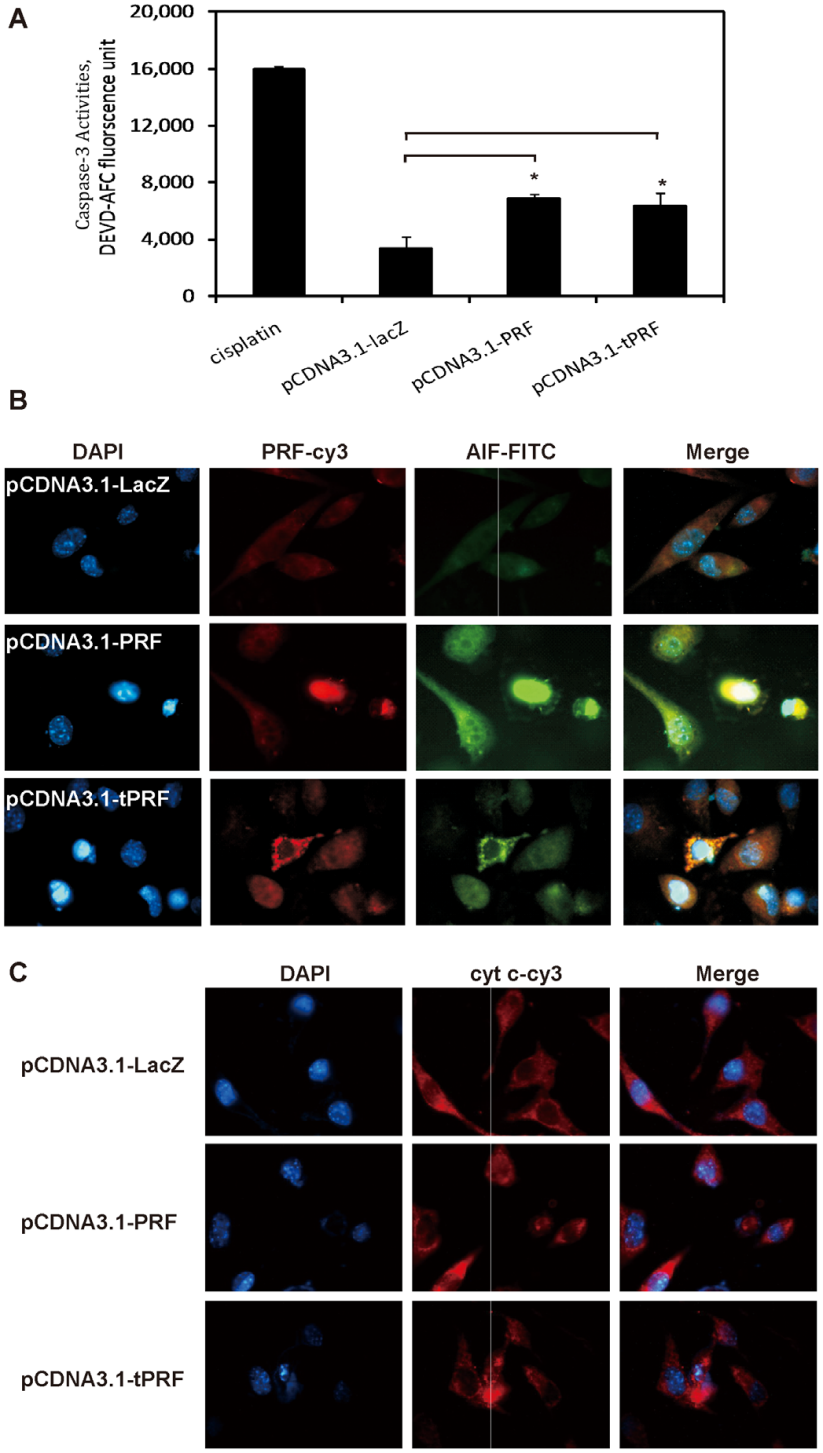
Effects of perforin on caspase-3 activity and release of AIF and cytochrome *c* from mitochondria. (A) Lysate of 1×10^6^ SK-BR-3 cells was collected 36 hours post transfection, and the intracellular caspase-3 activity was assayed with an ApoAlertTM Caspase-3 Fluorescent Assay Kit (BD, Protocol: PT3191-1) based on the release of free 7-amino-4-trifluoromethyl coumarin (AFC) by the activated caspase-3 cleavage of DEVD-AFC. Data are shown as mean ± S.D. of 3 experiments. *P* values were determined by unpaired Student’s t test (n = 3; * *P*<0.05). Cisplatin was taken as positive control. (B and C) SK-BR-3 cells after transfection were subjected to immunofluorescent staining using antibodies targeting to perforin, cytochrome *c* and AIF, and DAPI staining to indicate nucleus. For panel B, perforin (PRF) is labeled by Cy3 (red), AIF is labeled by FITC (green), and the nuclei is labeled by DAPI (blue); and panel C, cytochrome *c* (cyt c) is labeled by Cy3 (red) and the nuclei is labeled by DAPI (blue).

We then explored the underlying mechanism behind perforin-induced apoptosis. Caspase 3 activation plays a key role in initiation of cellular events during the early apoptotic process, and, accordingly, it has also been considered as a good marker to indicate apoptosis. Caspase-3 activity assay was performed 36 hours after the cells were transfected by perforins, and cisplatin was used as a positive control. The fluorochrome (free fluorescent AFC) generated by proteolytic cleavage of the caspase substrate (Ac-DEVD-AFC) was found to be proportional to the concentration of activated caspase 3 in the cell lysates after transfection. Cells transfected with both full-length and truncated perforin were found to have more caspase-3 activity than LacZ transfectants (Fig. 5A).

To determine potential toxic effects of perforin, the cellular distributions of AIF and Cyt c were detected by immunofluorescence staining. As shown in Figure 5B, the merged cy3 and FITC signals indicated that the expressed perforin facilitated AIF redistribution from the mitochondria to the nucleus. Figure 5C showed that Cyt *c* had also been translocated from the mitochondria to the nucleus. This process was characterized by apoptosis after transfection [26].All above indicated that the transfection of perforin may trigger both of the apoptotic pathway.

### Effects of Z-VAD and TAT-BH4 on Cell Death Induced by Ectopic Expression of Perforin

**Figure 6:**
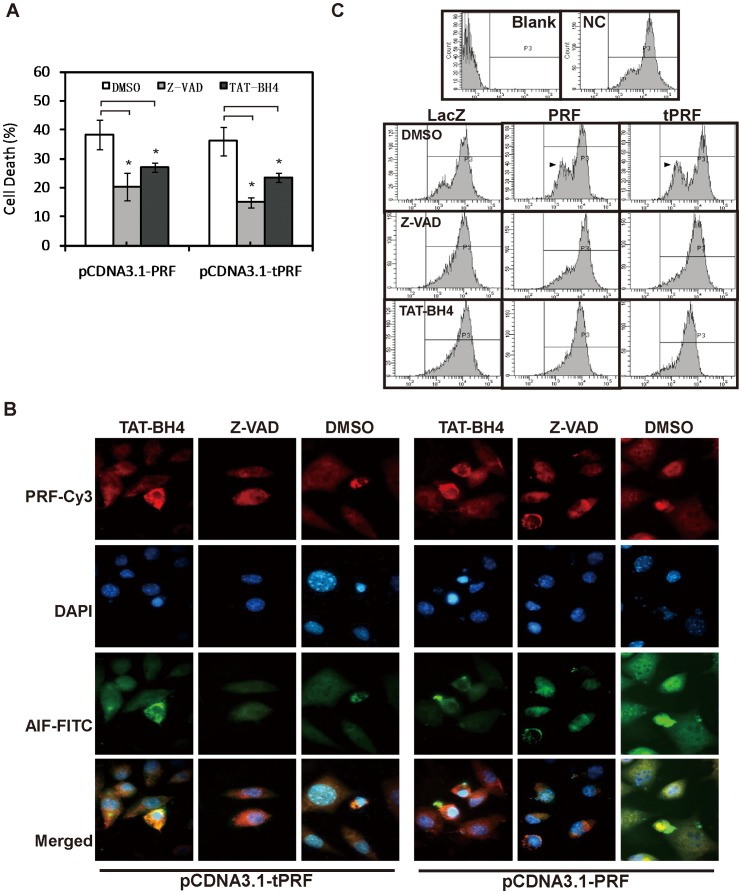
Effects of Z-VAD and TAT-BH4 on cell death induced by Ectopic expression of perforin. SK-BR-3 cells were transfected with full-length and truncated perforins in the presence of DMSO, Z-VAD(5 µM) or TAT-BH4 (100 nM). (A) Forty-eight hours later, cells were subjected to typan-blue staining and the light cells were counted. Cell death ratios were calculated by comparing Z-VAD or TAT-BH4 groups with DMSO group. Data are shown as mean ± S.D. of three experiments. *P* values were determined by unpaired Student’s t test (n = 3; * *P*<0.05). (B) Thirty-six hours later, cells were stained by anti-AIF and anti-perforin antibodies and observed under microscope. (C) Thirty-six hours later, cells were stained by DiOC6 and detected by flow cytometry. Blank spaces represent control cells untreated by DiOC6 dye and NC represents control cells dyed with DiOC6. Mitochondrial membrane potential losing is stressed by a black triangle.

To confirm that the apoptotic pathways promoted by cytosol perforin were involved not only caspase activation but also in the release of Cyt *c* and AIF from the mitochondria, pan caspase inhibitor (Z-VAD, Calbiochem) and a cell-permeable peptide that can inhibit the release of cytochrome c and loss of mitochondrial membrane potential (TAT-BH4, Calbiochem) were applied to the cells before transfection. As shown in Figure 6A, cell death induced by the expression of perforin was decreased in the presence Z-VAD (5 µM) and TAT-BH4 (100 nM). In addition, AIF was found involve the translocation of fewer nucleuses, as indicated by immunofluorescent staining (Fig. 6B). ΔΨ, which here represents the loss of mitochondrial membrane potential, was analyzed by flow cytometry using the cell-permeate green-fluorescent lipophilic dye DiOC6 (Molecular Probes, Eugene, OR, U.S.) which is actively taken up by intact mitochondria of living cells only. The data in Figure 6C show that, in the cells expressing perforin, less mitochondrial membrane potential was lost upon exposure to Z-VAD and TAT-BH4 than upon exposure to DMSO.

## Discussion

Studies have shown that perforin can initiate an internal suicide program in target cells via the well-characterized granule exocytosis mechanism [27]. The pro-apoptotic activity of the granzymes involved in this process has been studied extensively [28]. PRF pore formation has been shown to facilitate direct flux of proapoptotic granzymes into the cytosol [10], [21]. It has also been shown to trigger target cell membrane repair in the form of endocytosis, thus facilitating granzyme uptake [21]. During the process, perforin itself might also be transported into the cytosol and play a relatively independent role in the killing of target cells.

In order to understand other possible roles that perforin protein plays in the killing of target cells, we used an ectopic expression strategy to intensively study the human perforin gene.

Growth of both HepG2 and SK-BR-3 cells was inhibited, as indicated by cell-counting assay after either form of recombinant PRF was transiently transfected. Interestingly, from an alternative cell proliferation assay, although the viability of HeLa cells was greatly impaired after transfection (similar to HepG2 and SK-BR-3), neither form perforin affected the status of the Jurkat cells from which PRF was cloned. This T cell leukemia cell line may have some unknown protective mechanisms that render it insensitive to perforin toxicity [29], [30].

Growth inhibition and impaired cell cycle transition in perforin transfected cell lines indicated that 1) non-genetically regulated ectopic expression of perforin might be toxic to the host, even when its structure was intact and therefore suitable for trafficking and 2) the truncated form might become activated intracellularly and trigger cell death. If the expressed perforin undergoes accurate processing and trafficking, the membrane system of the host is first targeted without the CLs own initial self-protection mechanisms [29], [30]. Cell lysis would then be inevitable. On the other hand, if the active perforin could destroy from the inner side, other cell death pathways might also be involved.

The impaired cell cycle transition may indicate that the transfected cells are undergoing apoptosis. However, the cytolytic property of perforin should lead to necrosis. Cells that have been committed to apoptosis have exposed phosphatidylserine residues and therefore bind Annexin V. Propidium iodide (PI) cannot stain the DNA in the nucleus until cell membrane is compromised, which happens late in this process. Therefore, the Annexin-V-positive, PI-negative stained portion of the cell sample was taken to represent cells undergoing apoptosis. Cell lysis, however, leads to immediate compromise of the cell membrane and was here characterized by PI staining accompanied by phosphatidylserine residues binding to Annexin V. This was be used to distinguish the damaged and necrotic cells from the apoptotic cells. To determine what was happening in the PRF-transfected cells, morphologic features of HepG2 cells were further detected by electron microscopy. Nuclear shrinkage, DNA condensation, and the formation of apoptotic bodies, all of which are characteristic of apoptotic cell death, were clearly observed after both PRFs transfection, whereas membrane disruption and low electron density in the cytoplasm were exceptionally increased in pCDNA3.1-PRF transfectants. Similar results were obtained when the Annexin-V/PI dual stain was adopted on the same cell line or other cell lines such as SK-BR-3. The Annexin-V^+^PI^-^cells, which showed signs of early apoptosis, increased in number after treatment with both recombinant PRF, relative to controls. With respect to necrotic cell death (PI+), which was evaluated simultaneously, pCDNA3.1-PRF transfection exhibited more potent cytotoxicity, as reflected by a higher number of PI-positive cells portion (data not shown), whereas pCDNA3.1-tPRF mainly induced apoptotic cell death. This may be related to the absence of the signal peptide and inhibitory C-terminal. A recent study indicated that perforin pore structures can favor phosphatidylserine flip-flops, as measured by Annexin-V, at the concentrations that encourage granzyme-mediated apoptosis [31]–[32]. However, in our experiments, the apoptotic cell death induced by both full-length and truncated perforin was verified by TUNEL and caspase-3 activation assay.

Previous studies have shown that perforin is mainly involved in granzyme delivery [20], [32], and granzymes have been shown to be the sole determinants of apoptosis [33], [34], [35]. However, in the absence of granzymes in our ectopic expression system, perforin seemed to induce apoptotic cell death by itself. To examine the pathway involved in this process, key molecules of mitochondria apoptotic proteins (e.g., cytochrome *c* and AIF) were detected by indirect immunofluorescent staining of SK-BR-3 cells. Morphologic features under the fluorescence microscope signified that DNA condensation and nuclear distortion or disruptions reflected by DAPI staining were always accompanied by ectopic expression of either form of perforin. Of these, cytosolic and nucleic translocations of mitochondrial cytochrome *c* and AIF were notably increased.

Although mitochondrial pathway and caspase pathway were both activated, we tried to figure out which may be the major process. From the apoptosis initiation points of view, the apopotic pathway could be divided into the extrinsic one and the intrinsic one [36]. The extrinsic pathway usually bridged by transmembrane death receptor superfamily members, while the intrinsic pathway is integrate in developmental and stress cues through activation of a functional apoptosome which requires release of cytochrome c from mitochondria. Considering the pathway related molecules, we could find that the caspase activation was congenerous for both extrinsic and intrinsic apoptosis. Moreover, the mitochondrial molecules activation was unnecessary for caspase activation induced apoptosis; on the other hand, the mitochondrial pathway could be greatly enhanced by the activation of the apoptosome, and the sequencial caspases cascade [37], [38]. That was to say, if perforin could directly trigger caspases cascade, the mitochondrial pathway would be unnecessary involved, so only the caspases related inhibitor but not the mitochondrial related inhibitor would have effect on the process of cell death. On the contrary, if perforin triggered mitochondrial pathway at first, then both kind of the inhibitor could be helpful in rescue. Our experiment data finally showed that, when caspase inhibitors (pan caspase inhibitor z-VAD-fmk) or mitochondria protectors (TAT-BH4) [39] were independently administered before transfection, AIF translocation, nuclear damages, and overall cell death were all markedly suppressed in both groups. So we could safely predicted that the ectopic-expressed perforin interfere with the mitochondria to induce apoptotic cell death.

Although the ectopic expression strategy revealed a that perforin may have apoptosis-inducing activity, it may not have faithfully replicated what happens when a killer cell destroys a target cell in which perforin and granzymes have been co-delivered in the spatially limited region of the immune synapse. However, our results implicate a putative caspase and mitochondrial apoptotic pathway induced by perforin. The ectopic expression of full-length and truncated perforin might exert its toxicity during trafficking, as indicated by the fact that it traverses organelles that are rich in the phospholipids that can support its binding and oligomerization. The loss of the mitochondrial membrane potential and releasing of pro-apoptotic factor observed in perforin expressing cells suggested that the pore-forming activity of such a toxic molecule might target important cellular compartments. In this way, it must be restrained during processing and trafficking. The inner mechanisms that restrain this toxicity have yet to be defined.

## Materials and Methods

### Construction of Expression Plasmids

The human perforin (PRF) encoding cDNA was obtained by reverse-transcription PCR from total RNA derived from human Jurkat cells following stimulation with 100 IU/ml IL-2 (Sigma) for 24 hours using a Superscript Kit (Invitrogen). Primers P1 (5′-TTTGAATTCATGGCAGCCCGTCTGCTCCTC-3′) and P2 (5′-TTTTCTAGATTACCACACGGCCCCACTCCGGTT-3′) were used to generate the wild-type full-length human perforin (PRF). A truncated perforin (tPRF), with 21 and 48 amino acids deleted in the amino- and carboxy-terminal, respectively, was obtained by PCR using primers P3 (5′-TTTGAATTCGGATGGCCCCGTGCCACACA-3′) and P4 (5′-TTTGTCGACTTACTCATGGGAACCAGACTTGGG-3′). All the PCR products were subcloned into pcDNA™ 3.1/myc-His(-) A plasmid (Invitrogen) and confirmed by DNA sequencing. LacZ was constructed in to the same vector as a control for preclude the apoptotic effects of the over-expressed proteins.

### Cell Culture and Transfection

The human breast cancer cell (SK-BR-3), human hepatocellular carcinoma cell (Hep G2), human uterocervical carcinoma cell (HeLa), and human lymphoblast cell (Jurkat) were all purchased from ATCC (Manassas, VA, U.S.) and maintained in RPMI 1640 or DMEM (Invitrogen, Carlsbad, CA, U.S.), with 10% FBS or FCS with 4 mM L-glutamine and adjusted to contain 1.5 g/L sodium bicarbonate at 37°C and 5% CO_2_. Transfection of cells was performed with Lipofectamine 2000 (Invitrogen), as described. Opti-MEM Reduced Serum Medium without serum (Invitrogen) was used to dilute DNA or Lipofectamine 2000. In some of the experiments, cells were incubated with the broad-spectrum caspase inhibitor N-benzyloxycarbonyl-Val-Ala-Asp-fluoromethyl-ketone at the final concentration of 5 µM (Z-VAD, Calbiochem), or with a cell-permeable peptide that contains the conserved N-terminal homology domain (BH4) of Bcl-xL linked to the HIV-TAT sequence at the final concentration of 100 nM (TAT-BH4, Calbiochem). DMSO, z-VAD, or TAT-BH4 was added to the growth media 1 h before transfection. The growth medium was not changed before the following assays.

### Cell Proliferation Assay

Cells were seeded (0.5×10^5^ for Hep G2 and 0.5×10^4^ for SK-BR-3) into 24-well plates in 500 µL of culture medium and incubated at 37°C, 5% CO_2_. After transfection for various times, cell numbers and viability were determined by direct counting with trypan blue exclusion as follows: a) Detachment of the adherent cells with 0.25% (m/v) trypsin (1∶250) at 37°C. b) Resuspension of cells in DMEM containing 10% FBS at a certain volume. c) Staining of the cells with Trypan Blue to the final concentration of 0.04% which was used to tell the dead cells from the living cells. d) Dropping the resuspended cells into the blood count plate. e) Counting the unstained cells in 4 squares, each containing 16 smaller squares. f) Calculating the cell concentration to be [(N1+N2+N3+N4)÷4]×10^4^/ml. g) The cell number is the result of the cell concentration times the total volume of the resuspended liquid and Trypan Blue used.

### 3-(4,5-dimethyl-thiazol-2yl)-5-(3-carboxymethoxyphenyl)-2-(4-sulfophenyl)-2*H*- Tetrazolium Assay

To estimate cell proliferation, cells were seeded (2×10^3^) into 96-well plates in 100 µL of culture medium and incubated at 37°C, 5% CO_2_. After culturing for various times, 20 µL of Cell Titer 96 Aqueous One Solution (Promega, Madison, WI, U.S.) were added to each well and cells were then incubated for 1 h in a 37°C, 5% CO_2_ incubator. To stop the reaction, 25 µL of 10% SDS was added and absorbance was measured at 490 nm.

### Western Blot Analysis

Cells were cultured in their respective medium in 10 cm culture dishes to 70–80% confluence (5×10^6^) and harvested 24 h after transfection in 200 µL of RIPA buffer (1×PBS, 1% Nonidet P-40, 0.5% sodium deoxycholate, 0.1% SDS, 1 mmol/L Na_3_VO_4_, 1 mmol/L aprotinin, and 1 mmol/L phenylmethylsulfonyl fluoride). The samples were disrupted by sonication and cell lysate clarified by centrifugation at 12,000 rpm for 10 min. The protein concentration was determined using the Bradford method [40]. The samples (30–50 µg protein) were resolved using 10% SDS polyacrylamide gel electrophoresis (SDS-PAGE) and subsequently transferred onto an Immobilon-P transfer membrane (Amersham Biosciences). The membrane was then incubated with primary antibody recognizing perforin (1∶200 in PBST, Santa Cruz) overnight at 4°C. After incubation with horseradish peroxidase-conjugated secondary antibody (1∶2000, ZhongShan), Western-blots were visualized using an enhanced chemiluminescence kit (Pierce).

### TUNEL Assay

For TUNEL assay, samples were cultured on cover slips, then fixed with 4% paraformaldehyde in PBS and post-fixed with ice-cold ethanol/acetic acid (2∶1) solution. After washing with PBS, the samples were treated with proteinase K (20 mg/ml in PBS) for 15 min at RT. The samples were then washed with PBS again and processed using a Fluorescein FragEL™ DNA Fragmentation Detection Kit (Calbiochem) according to the manufacturer’s instructions. The samples were also counter-stained with DAPI. To measure the rate of apoptotic cell death, we measured the TUNEL-positive cell from photographs of the stained samples (400× magnification) by counting of TUNEL-positive cells in 4 areas with an average cell number of 200 cells that were grown on cover slips.

### Immunofluorescence Staining

The cells were cultured on cover slips and then fixed with a freshly prepared paraformaldehyde solution (4% in PBS, pH 7.4) for 10 min at room temperature. They were then permeabilized with 0.01% Triton X-100 for 10 min on ice. They were detected with primary antibodies recognizing perforin (1∶400; Neo Marker) and AIF (1∶400; PharMingen). They were then treated with biotin-linked anti-mouse IgG (1∶200; Boshide) as secondary antibodies and Cy3-linked avidin (1∶200; Boshide) as the tertiary reagent, or FITC-linked anti-goat IgG (1∶200; Boshide) as the secondary antibodies. DAPI (Calbiochem) was further used for nucleus staining. The cells were observed with a fluorescence microscope.

### Substrate Cleavage Assay

To assay for caspase-3 activity *in vitro* (ApoAlertTM Caspase-3 Fluorescent Assay Kit; BD), 1×10^6^ SK-BR-3 cells were resuspended in 50 µl chilled cell lysis cbuffer and incubated on ice for 10 min. Cell lysates were centrifuged and the supernatants transferred to a new tube with 50 µl 2×reaction buffer/DTT mix. In each reaction, 5 µl of 1 mM caspase-3 substrate (DEVD-AFC) was added to a final concentration of 50 µM. After incubation for 1 h at 37°C, the AFC liberated from the Ac-DEVD-AFC was measured with a spectrofluorometer with an excitation wavelength of 400 nm and an emission wavelength of 480–520 nm (peak, 505 nm).

### Assessment of Apoptosis by Monitoring Exposure of Phosphatidylserine

Cells (1×10^6^ cells) after transfection were washed with PBS twice and resuspended in binding buffer containing Annexin-V-FLUOS and propidium iodide (Annexin-V-FLUOS Staining Kit; Roche Applied Science) and stained for 10 min at RT. Subsequently, the cells were analyzed by flow cytometry.

### Assessment of Apoptosis by Monitoring Loss of Mitochondrial Membrane Potential (ΔΨ)

After treatment, cells were harvested by centrifugation (1,000×*g*, 5 min), incubated for 30 min at 37°C with fresh medium containing 0.1 µmol/L DiOC6 (Molecular Probes, Eugene, OR, U.S.), washed once with PBS, and analyzed by flow cytometry.

## Supporting Information

Figure S1 **Growth curves of perforin transfected Jurkat cells.** Growth curves of transfected Jurkat cells were assessed by a MTT assay. Data are represented as mean ± S.D. of 3 experiments.
(TIF)
