# Supplementary figures and images for: Ectopically Expressed Perforin-1 Is Proapoptotic in Tumor Cell Lines by Increasing Caspase-3 Activity and the Nuclear Translocation of Cytochrome c
Source: PLoS One. 2012 Jul 19;7(7):e40639. doi: 10.1371/journal.pone.0040639 (PMC3400674; doi:10.1371/journal.pone.0040639)

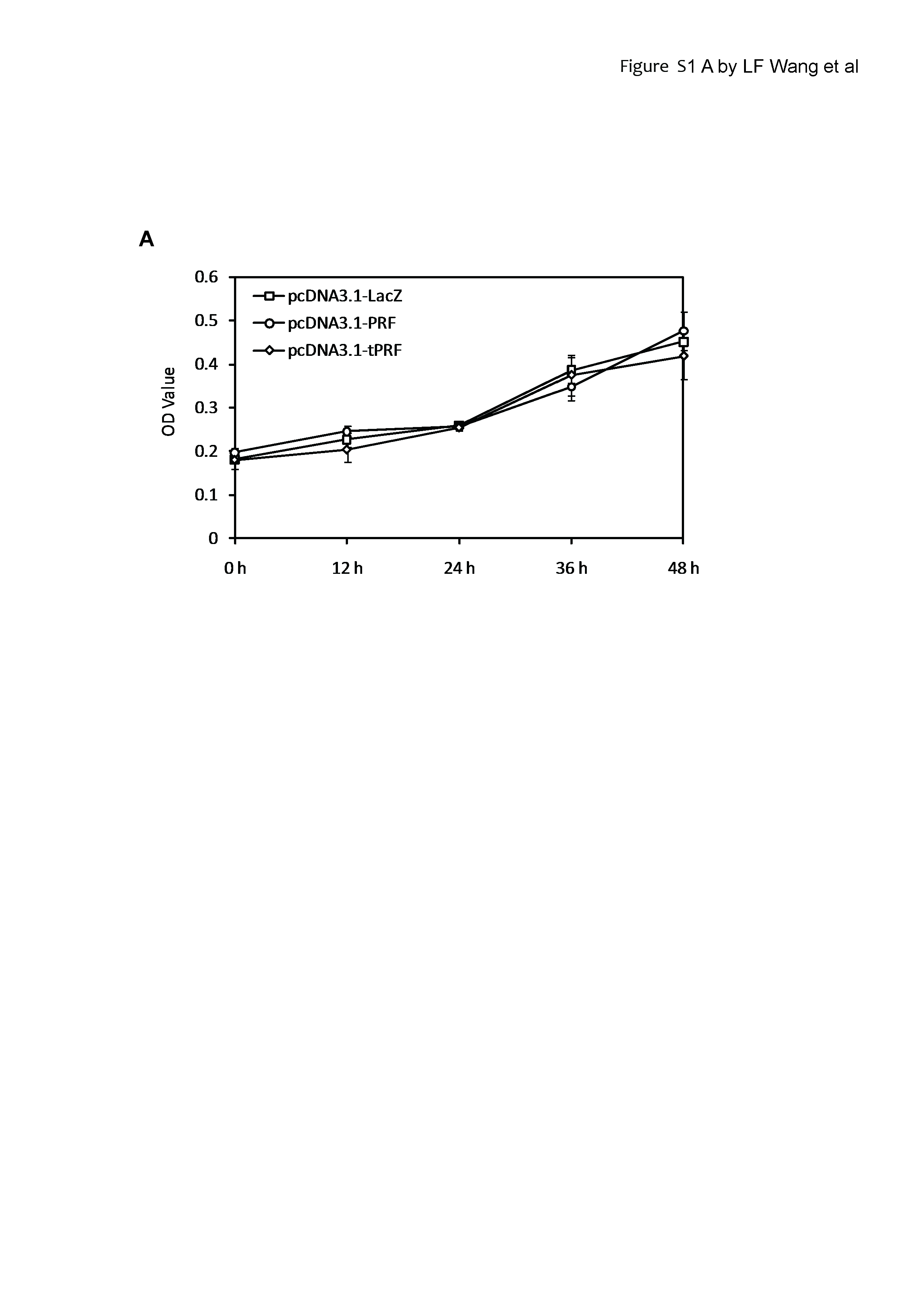

Supplement: Figure S1 — Growth curves of perforin transfected Jurkat cells. Growth curves of transfected Jurkat cells were assessed by a MTT assay. Data are represented as mean ± S.D. of 3 experiments. (TIF) [file pone.0040639.s001.tif]
